# Contrasting Fish Behavior in Artificial Seascapes with Implications for Resources Conservation

**DOI:** 10.1371/journal.pone.0069303

**Published:** 2013-07-30

**Authors:** Barbara Koeck, Josep Alós, Anthony Caro, Reda Neveu, Romain Crec'hriou, Gilles Saragoni, Philippe Lenfant

**Affiliations:** 1 Univ. Perpignan Via Domitia, CEntre de Formation et de Recherche sur les Environnements Méditerranéens, Perpignan, France; 2 CNRS, CEntre de Formation et de Recherche sur les Environnements Méditerranéens, Perpignan, France; 3 Instituto Mediterráneo de Estudios Avanzados, IMEDEA (CSIC-UIB). C/Miquel Marqués 21, Esporles, Illes Balears, Spain; The Australian National University, Australia

## Abstract

Artificial reefs are used by many fisheries managers as a tool to mitigate the impact of fisheries on coastal fish communities by providing new habitat for many exploited fish species. However, the comparison between the behavior of wild fish inhabiting either natural or artificial habitats has received less attention. Thus the spatio-temporal patterns of fish that establish their home range in one habitat or the other and their consequences of intra-population differentiation on life-history remain largely unexplored. We hypothesize that individuals with a preferred habitat (i.e. natural vs. artificial) can behave differently in terms of habitat use, with important consequences on population dynamics (e.g. life-history, mortality, and reproductive success). Therefore, using biotelemetry, 98 white seabream (*Diplodus sargus*) inhabiting either artificial or natural habitats were tagged and their behavior was monitored for up to eight months. Most white seabreams were highly resident either on natural or artificial reefs, with a preference for the shallow artificial reef subsets. Connectivity between artificial and natural reefs was limited for resident individuals due to great inter-habitat distances. The temporal behavioral patterns of white seabreams differed between artificial and natural reefs. Artificial-reef resident fish had a predominantly nocturnal diel pattern, whereas natural-reef resident fish showed a diurnal diel pattern. Differences in diel behavioral patterns of white seabream inhabiting artificial and natural reefs could be the expression of realized individual specialization resulting from differences in habitat configuration and resource availability between these two habitats. Artificial reefs have the potential to modify not only seascape connectivity but also the individual behavioral patterns of fishes. Future management plans of coastal areas and fisheries resources, including artificial reef implementation, should therefore consider the potential effect of habitat modification on fish behavior, which could have key implications on fish dynamics.

## Introduction

Since the 1980's, artificial reefs (AR) have been increasingly used as management tools, mainly to offset marine resource declines and enhance fish production [1], [2], [3]. In contrast with other, more widely used management tools, such as fishing quotas [4] and marine protected areas [5], [6], the deployment of ARs induces a physical alteration of bottom substrate, generally with the replacement of the naturally present soft bottom by concrete structures [7]. The deployment of these ARs adds to the other already existing habitat modifications of coastal areas induced by ongoing urbanization, such as the construction of harbors, seawalls, breakwaters or pontoons [8]. In contrast to habitat loss or degradation, these newly created habitats diminish inter-patch distances [9] generating artificial seascapes. These newly added hard-bottom habitats alter seascapes both by their habitat structure (i.e. habitat complexity, heterogeneity and nature) [10], and by their habitat edges [11]. These modifications of habitat structure and edges have the potential to act on communities, as well as on the diversity and abundance of species [12], [13] and species interactions by restricting or facilitating the movement of organisms within a seascape [11], [14]. For example, it is known from coral reef studies that, on the scale of a reef, sand gaps act as partial barriers to fish movements [15], [16]. Habitats with low complexity are known to provide fewer opportunities for refuge and to incur increased predation risks [17], [18], [19]. ARs immersed on these sandy gaps will probably change habitat utilization and spatio-temporal fish population dynamics.

Nevertheless, the comparison between the behavior of wild fish inhabiting natural and artificial habitats has received less attention [20] and the spatio-temporal patterns of fish that establish themselves in one habitat or the other remain unexplored. One of the main causes of this lack of study is the difficulty to track and monitor individual fish in the wild. However, the continuous technical improvement of electronic tags has greatly contributed to the advances in our understanding of the interaction of species and their environment [21], [22]. Specifically, acoustic tagging in marine ecosystems has enabled remote monitoring of animal movements and has thus provided insight into the spatial dynamics of many marine animals [23], [24], even for highly mobile fish [25], [26]. Changes in diel behavior for several temperate reef fish can notably be inferred from the analysis of acoustic detection patterns [27], [28], some of which are due to the behavior of hiding in rocky crevices [29] or seagrass meadows [30], [31] during periods of rest. Moreover, the continuous development of statistical approaches to derive behavioral patterns from telemetry data has been improved during the last years [32], notably the utilization of wavelet analysis to draw temporal patterns from movement data [33]. Therefore, the technical improvements of electronic tags coupled to the innovation and development of powerful tools for analyzing the data provide a suitable context in which to study behavioral differences at the scale of small seascapes dominated by natural and artificial habitats.

In this study, we focused on the white seabream, *Diplodus sargus* (Linnaeus, 1758), a widely distributed and abundant demersal Sparidae of the Mediterranean and eastern Atlantic. This species is mostly found on rocky and vegetated bottoms [34], [35] at depths mainly less than 50 m [36]. Nevertheless, this species also occurs on sandy bottoms and lagoons. White seabream reach first maturation at around 17 cm [37] and is generally considered as a rudimentary hermaphrodite with partial protandry [38], [39]. This species is omnivorous, feeding mostly on benthic invertebrates but also on seaweeds [40]. FAO fisheries statistics [41] show a constant increase of global white seabream catches, which mainly correspond to artisanal fisheries. Throughout the study area, the white seabream is also a locally important commercial species, which accounts for about 7% of the catch by weight for artisanal fisheries [42]. A previous study carried out on the ARs of Leucate – Le Barcarès showed high abundance using underwater visual census [43]. High abundances of white seabream were also reported on ARs on the Portuguese coast [44] and the Italian coast in Sicily [45], highlighting that ARs are potentially suitable habitats for this species. Moreover, D'Anna *et al.* [45] studied the movement patterns of wild white seabream inside and in the close vicinity of AR sets. According to this study, white seabream showed strong site fidelity to AR and nocturnal movement patterns, hiding inside the AR set during the day and searching for food around the artificial structures by night. The white seabream is therefore a good candidate for exploring behavioral differences and their implications on individuals inhabiting both natural and artificial habitats.

In an ongoing context of coastal habitat alteration [46], it appears essential to take into account these newly created habitats to better understand the movement patterns of fish and the implications of ARs on the management of marine coastal resources. Based on the hypothesis that individuals inhabiting one habitat or the other can behave in a different manner in terms of time and spatial habitat use, the main aim of this study was to compare movement patterns of white seabreams inhabiting an artificial reef (AR) system with those of the closest natural rocky reef. Prior to that, white seabreams were categorized into behavioral pattern groups using individual detection rates and residency indexes per habitat type. In order to distinguish between the effect of habitat type and the effect of individual adaptation on the temporal diel pattern, we tested diel detection patterns by pooling detections (i) by habitat types and (ii) by fish with the same behavioral pattern. Furthermore, inter-habitat movement patterns were assessed to evaluate the implications of ARs in functional seascape connectivity.

## Materials and Methods

### 1. Study area

**Figure 1:**
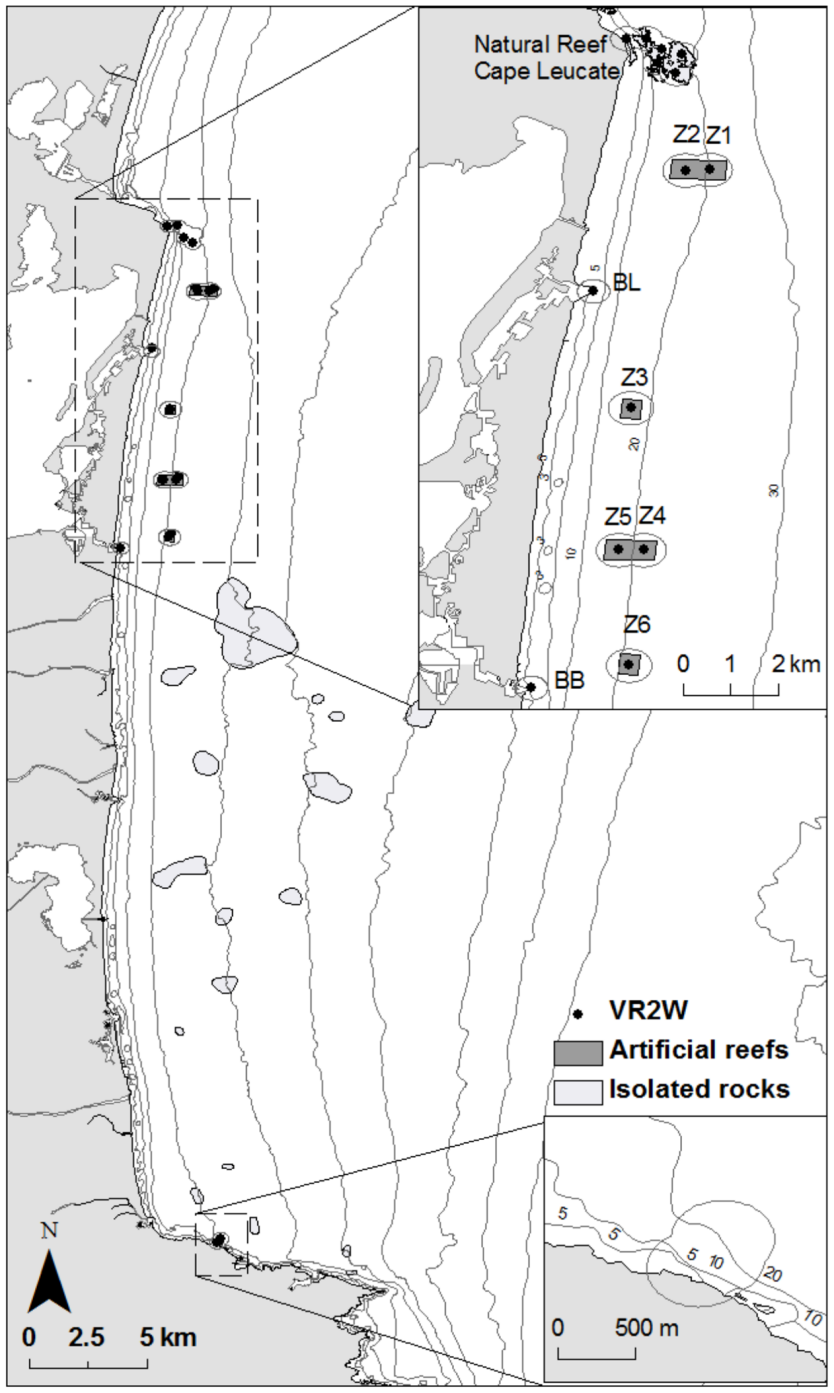
Study area and acoustic receiver locations. Grey ellipses around the VR2W receivers correspond to their average detection range (250 m on the NR and 350 on the AR).

The Leucate – Le Barcarès ARs are located along the French Catalan coast, in the northwestern Mediterranean Sea (Figure 1). This AR system is located along a sandy coastline, but surrounded by both natural rocky and artificial habitats and a lagoon. The sandy Catalan coast is bordered by different rocky habitats: in the north by the Cape Leucate (CL) and in the south by the ‘Côte Vermeille’ (CV), at respectively 8 and 35 km from the center of the studied AR. This highly urbanized coast also presents artificial structures like seawalls, breakwaters and the studied AR-system off the coast between Leucate and Le Barcarès. This AR system was immersed in 2004 on a sandy substrate and is composed of 6 reef groups named Z1 to Z6. These AR groups run parallel to the coastline along the 15 and 25 m isobaths (Figure 1) and each consists of 28 sets of concrete reefs. Reef groups Z2, Z3 and Z5 are 15 to 19 m deep, and Z1, Z4 and Z6 are more than 20 m deep. Reefs sets are placed 50 m apart, with an AR group occupying a total area of 120 000 m^2^.

### 2. Fish collection and intracoelomic tagging

#### Ethic statement

Under the French legislation concerning experimentation on vertebrates, the capture, handling and tagging procedures were approved by the departmental direction of populations and animal protection DDPP (certificate number A66–12–01) and the French Ministry of Agriculture (approbation n°R-21UB/EPHE-F1-11). No protected species were sampled in this study. Tagging was performed under anaesthesia, and all efforts were made to minimize suffering.

The white seabreams used in this study were caught by a professional fisherman using long-lines. To ensure that the fish were healthy for the tagging experiment, long-lines were never set for more than one night. The fish caught were brought to land in an oxygenated tray and directly tagged in the harbor to avoid additional stress through transport and captivity. The tagging procedure was performed according to the methodology described in Koeck *et al.* [47]. In short, the fishes were individually anaesthetized, measured, weighed and tagged with externally visible T-bar anchor tags to be returned in case of recapture by fishermen [47]. A coded acoustic transmitter from VEMCO (AMIRIX Systems, Canada, Halifax) was inserted into the coelomic cavity and sutured with non-absorbable polyamide monofilament and a curved cutting needle. We used V9-2L transmitters (power output: 146 dB, weight: 4.7 g in air, dimensions: 29 mm x 9 mm, battery life: 151 days), programmed with an average ping interval of 60 sec (45 to 95 sec). Previous tests have shown that this ping interval is adapted to the monitoring of white seabream movement patterns with the present acoustic receiver array, avoiding the loss of important detections for the interpretation of their movement pattern due to a too high signal interval (unpublished data). After surgery, fish were kept in an oxygenated 50-L tray until they fully recovered, i.e. until equilibrated swimming was recovered [48], [49]. In order to avoid mortality after release or monitoring of biased movement patterns due to injuries caused during the tag implantation, fish which did not recover fully within 20 min were removed from sampling [47].

Altogether, 98 fish were tagged and released at their capture site between May and October 2011, 42 of which were released at the natural reef of Cape Leucate (NR) and 56 at the AR sites (AR). As reviewed in Davis [50], stress due to manipulation and tagging can cause delayed fish mortality and represent an important issue in tagging experiments. Koeck *et al.* [47] demonstrated however that intracoelomic tagging has no adverse effects on white seabream behavior and survival. Veiga *et al*. [51] showed also that post-release hooking mortality of *Diplodus vulgaris*, which is a close species of *D. sargus*, tends to be very small. Nevertheless in the present study, hooked fish were exposed to particularly strong local currents between the 30th May and 22th June 2011, which could have compromised their condition and ability to recover from tagging. Therefore, fish tagged during this period with a detection period below 20 days and detected only by the receiver from release location were supposed to be dead and thus excluded from the analysis (24 fish).

### 3. Long-term passive monitoring

An array of 17 VEMCO VR2W acoustic receivers was deployed for the long-term continuous monitoring of the movements of white seabream. These receivers were able to record nearby acoustic tags emitting at a frequency of 69 kHz. For each detection, they register date and time and the unique ID of the received transmitter. The deployment strategy of receivers was implemented according to our current knowledge of the habitat preferendum of white seabream, by covering the main rocky bottoms of the study site, but also the six reef groups of the surveyed ARs and the closest seawalls (Fig. 1). Thus, six VR2W were deployed on Cape Leucate, one VR2W in the center of each AR group on the top of a culvert box reef (a total of 6 receivers on ARs), one at the end of the Leucate harbor seawalls and Le Barcarès harbor seawalls. These two harbor seawalls also correspond to two of the three channels communicating with the lagoon of Salses-Leucate.

According to water depth, two different attachment systems were used to anchor the receivers to the bottom. In waters above 8 m depth, receivers were moored in a PVC-pipe vertically sunk into a concrete-filled tire. On the ARs, the tires were additionally hooked at each end of the culvert-box. In shallow waters (below 8 m depth), receivers were attached to steel torches that were sunk in cement between the rocks. The detection probability of acoustic signals can vary greatly depending on substrate type, sea state, water depth and turbidity [52], [53], [54], [55]. Detection range tests were thus performed on the two different studied habitats where receivers were anchored: Cape Leucate with a shallow rocky coast (3 to 25 m depth) and the Leucate – Le Barcarès ARs which are surrounded by sandy bottoms (15 to 25 m depth). According to the range tests, the mean detection radius of the ARs was 350 m and 250 m for the NR.

### 4. Standardization of sampling effort

The sampling effort of fish movement between NR and AR habitats was unbalanced due to the differential detection coverage and extent of these habitats. Thus, detection densities were standardized by detection surface (detections per km^2^). The detection areas for each habitat were calculated according to receiver number, detection range of the habitat and the overlapping of the detection ranges of some receivers. Sampling areas were 1.69 km^2^ for the ARs and 0.75 km^2^ for the NR.

As recently mentioned by Payne *et al.* [54], the probabilities of the detection of acoustic waves in coastal waters are highly variable. They depend on environmental noise, due to wave action [53], [56], biological activity [57] or on physical impediments, like increased water turbidity or thermoclines [58], [59]. To account for the temporal variability of the detection probabilities in the NR and AR habitats, we deployed three control tags in each habitat at a fixed position during the study period (i.e. three tags deployed between the two AR groups Z4– Z5 and two receivers in the middle of the CL at distances ranging from 100 to 450 m). These control tags were chosen to have the same power output and dimensions as the tags used for the fish, except that the delay between two signals was chosen much lower (9 min delay) to avoid signal collision between tags. If a temporal pattern in the detection probabilities of control tags was detected, a correction of the detections of tagged fish by control tag detections was applied according to the method described in Payne *et al.* [54]. This method consists in calculating a corrective coefficient from the mean detection probability of control tags by hourly bin. Our study being conducted over several months, and assuming that these probabilities could vary with episodic or seasonal events, we computed and applied this corrective coefficient on a weekly basis rather than for the overall study period.

### 5. Data analysis

Among the 74 fish kept for the analyses, 36 were caught and released at the NR and 38 at the AR locations. These remaining fish were then grouped into behavioral groups according to their residency indexes for each habitat type (ratio between days spent in each habitat and overall detection days) and general detection rates (ratio between days detected and overall detection period).

Prior to data analysis, given the large number of transmitters used in this study enhancing the risk of false detections due to signal collision, single daily detections were removed from the dataset. Visual inspection of chronogram plots (hourly detection number over the entire detection period) and continuous wavelet transform (CWT) were used for the temporal analysis of individual transmitters (fish and control tags). CWT analysis is an alternative method to Fast Fourrier Transform (FFT) or other time-frequency decomposition methods to test periodicities over different time-scales [33]. Two-dimensional wavelet spectrums and point-wise tests at a significance level of 95% were performed for each transmitter. Mean temporal patterns of control tags and fish detections pooled by habitat type (AR, NR) and pooled by behavioral fish group were visually inspected using plots of mean detections per hourly bin and then formally tested with a generalized linear mixed effects model (GLMM) using a Poisson distribution. GLMM is a suitable technique for the analysis of non-normal data with random effects [60]. In our case, data was non-normally distributed even after transformation efforts and concerned repeated measures (detections) of transmitters and days. Days and transmitter (fish or control tags) were thus considered as random factors. The diel phase and size of fish were treated as fixed factors. GLMM parameters were estimated using a Laplacian approximation. The normality of residuals and model performance were visually examined using residual distributions and quantile-quantile plots of residuals against fitted values. The correlation between fish and control tag detections for the NR and AR were tested using a Pearson's correlation test. Size distribution of fish was tested using a one-way ANOVA between the different identified behavioral groups, to ensure it is not biasing diel movement pattern results. Detections were categorized into day and night phases according to sunset and sunrise data from the US Naval Observatory (http://www.usno.navy.mil/USNO/astronomical-applications/data-services). CWT analyses were performed using the SOWAS-package [61] and GLMM using the lme4-package [62] computed for the R statistical environment [63]. The spatial patterns of the tagged white seabream were compared by visual inspection of the plots of mean detections per hourly bin for each receiver, of the number of total receivers detected and of the number of excursions outside the preferred habitat (i.e. number of visits to other habitats; a visit consisted of a series of hourly detections without being detected on the preferred habitat).

## Results

### 1. Control tags: removal of environmental variability from acoustic detections

**Figure 2:**
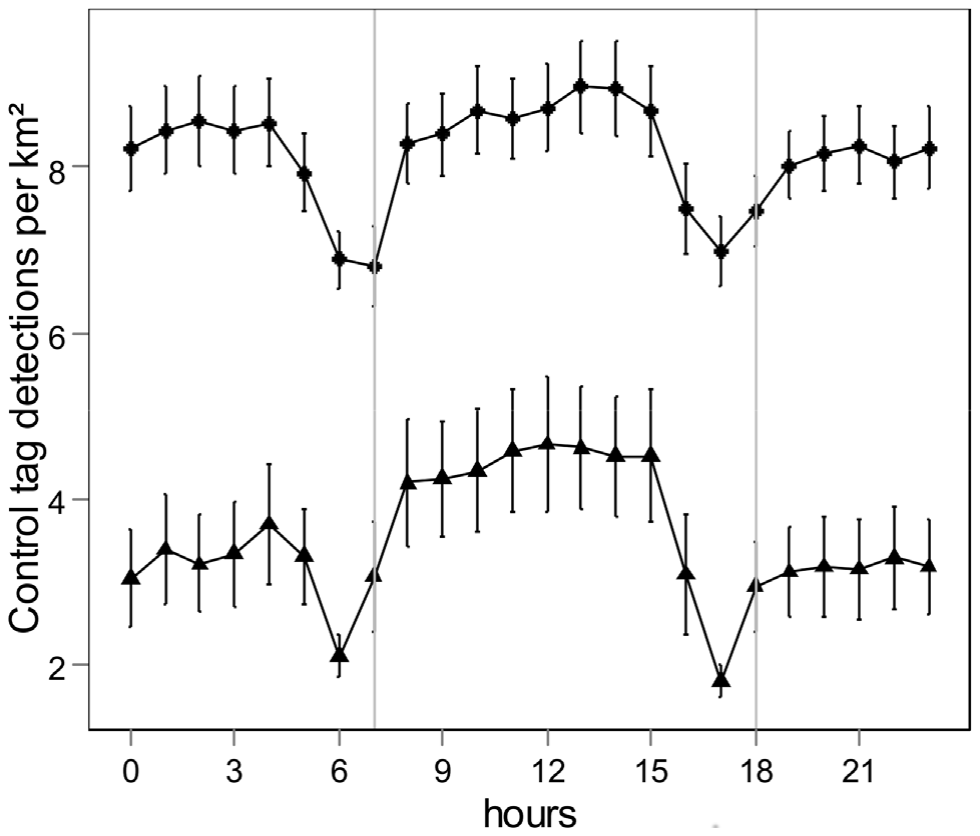
Mean detections and standard errors per hourly bin of control tags on the ARs (circles) and on the NRs (triangles). Detections are standardized by sampling surface and thus expressed per km^2^. Vertical grey lines symbolize mean sunset and sunrise over the entire study period.

**Figure 3:**
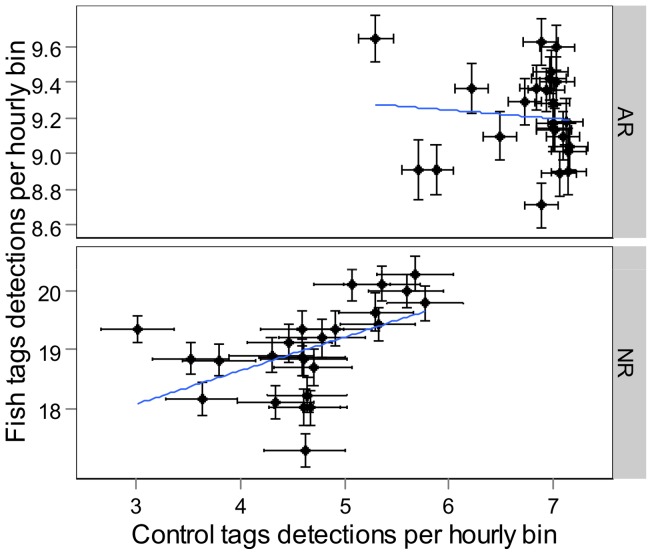
Correlation plots between control tag detections and raw detections of tagged white seabreams on the artificial reef (AR) and the natural reef (NR).

Mean detections per hourly bin of control tags varied over the day both for the ARs and the NR (Fig. 2). The AR and NR control tags showed a similar pattern with a sharp decrease in detections around the sunrise and sunset hours and slight differences in detections between day and night. The CWT analysis highlighted no significant temporal periodicity for control tags over the entire detection period. However, GLMM analysis showed significant differences in detections between day and night for NR control tags (Table 1), with higher detections during the daylight hours. No differences were detected for the AR control tags. These results suggest that the observed temporal detection patterns were influenced by environmental factors, at least on the NR. The correlation test between raw fish detections and control tag detections for the AR and the NR showed that there was no correlation between hourly bin detections for the AR (R^2^  = −0.9, p-value  = 0.6698; Fig. 3). In contrast to the NR, there was a low correlation between control tag and raw fish detections (R^2^  = 0.52; p-value  = 0.0124; Fig. 3). These results confirmed those from the temporal analysis of control tags: detection probabilities on the NR are influenced by environmental factors. As the correlation between raw fish detection and control tags on the NR is rather weak, it also highlights that temporal fish patterns are not only due to environmental factors, but that there is also a behavioral pattern of fish that should be visible with the analysis of fish detections corrected by the diel detection pattern of control tags. For further analyses, fish detections were thus corrected by the detections of control tags of the corresponding habitat (AR or NR) to remove the environmental effect from the observed pattern.

**Table 1:** Results of generalized linear mixed models testing the effect of diel phase (day vs. night) on mean detections of control tags on artificial reef (AR) and natural reef (NR).

	estimate	SE	z-value	Pr(>|t|)	
***a. AR control tags***					
intercept	1.988	0.051	38.87	<2e-16	***
diel phase (day)	−0.009	0.006	−1.51	0.131	ns
***b. NR control tags***					
intercept	0.878	0.495	1.77	0.076	ns
diel phase (day)	0.159	0.016	9.55	<2e-16	***

‘ns’ p>0.05, ‘*’ p<0.5, ‘**’ p<0.01, ‘***’ p<0.001.

### 2. Residency and fish movement pattern groups

The tagged white seabream ranged between 17 and 35 cm standard length, with a mean size of 25.91±5.6 cm. Weight ranged between 155 and 1220 g with a mean value of 596.96±34.96 g. The mean detection period was of 113+/−67 days and reached 9 months for some individuals. Detection rates were highly variable between individuals, ranging from 4 to 100% of days detected (Table 2). Inspection of global detection rates and residency indices of each habitat type of each tagged white seabream permitted the distinction of four behavioral groups. A first group of 15 fish (20% of tagged fish) was characterized by low detection rates (DR <50%) conveying an occasional usage of hard bottoms, and were called transient individuals (T). The three other groups correspond to individuals with high detection rates (DR >70%), 2 groups of which presented a clearly identified preferred habitat. On the one hand there were 20 AR-resident fish (27%) with a preference for the AR habitat (RI_AR_  = 70%), and on the other hand there were 33 NR-resident fish (45%) with a preference for the Cape Leucate NR habitat (RI_NR_  = 94%). The last group consisted of 6 fish (8%) with No Preferred Habitat (NPH), with high detection rates but which switched from AR to NR during the monitoring period (Table 2). We noticed that out of the 20 AR-resident fish one was captured on the NR. Out of the 33 NR-resident fish, three were captured on the northern ARs and one on the seawall of Leucate (SW- BL). All fish of the NPH group were tagged on the ARs. Transient fish (TR) equally consisted of fish tagged on the NR and AR, with one fish tagged to the SW of Leucate.

**Table 2:** Information of individual tagged white seabreams concerning their capture, their residency and movement patterns.

Fish ID	Capture location	Movement Group	Standard length (cm)	Weight (g)	Date released	DP (days)	DR	RI (NR)	RI (AR)	RI (SW)	RI (CV)	EOPH
1	AR	AR	26.0	590	30/07/2011	91	98.90	0.00	100.00	0.00	0.00	0
2	AR	T	31.2	1005	30/07/2011	155	12.26	96.34	3.66	0.00	0.00	1
3	AR	NPH	33.5	1220	30/07/2011	106	86.79	51.64	48.36	0.00	0.00	3
4	AR	NPH	34.3	1125	29/07/2011	209	96.17	54.50	45.48	0.02	0.00	41
5	AR	T	32.4	1050	29/07/2011	72	4.17	26.67	60.00	0.00	13.33	1
6	AR	AR	24.7	480	29/07/2011	150	98.67	28.91	71.03	0.00	0.06	2
7	NR	NR	21.6	330	22/06/2011	22	95.45	100.00	0.00	0.00	0.00	0
8	NR	NR	31.6	985	22/06/2011	138	78.26	99.58	0.27	0.00	0.15	6
9	AR	NR	31.3	915	22/06/2011	27	96.30	98.97	1.03	0.00	0.00	1
10	AR	NPH	28.2	650	29/07/2011	68	100.00	44.23	55.77	0.00	0.00	1
11	AR	NR	23.6	385	21/06/2011	26	92.31	99.85	0.15	0.00	0.00	1
12	AR	AR	34.6	1140	21/06/2011	114	98.25	0.00	100.00	0.00	0.00	0
13	NR	NR	28.8	795	21/06/2011	52	100.00	100.00	0.00	0.00	0.00	0
14	NR	NR	29.1	750	22/06/2011	196	77.65	99.90	0.10	0.00	0.00	1
15	NR	NR	33.0	1045	22/06/2011	148	97.97	99.88	0.12	0.00	0.00	2
16	AR	T	19.3	225	16/06/2011	38	47.53	0.00	3.41	42.64	53.94	1
17	NR	NR	26.8	650	17/06/2011	201	94.53	99.88	0.12	0.00	0.00	2
18	NR	NR	23.6	420	17/06/2011	41	100.00	100.00	0.00	0.00	0.00	0
19	NR	NR	27.2	635	17/06/2011	193	72.69	100.00	0.00	0.00	0.00	0
20	AR	AR	23.2	395	21/06/2011	248	96.37	0.00	100.00	0.00	0.00	0
21	AR	AR	30.9	835	18/06/2011	207	90.34	21.54	78.46	0.00	0.00	1
22	AR	NPH	26.9	650	16/06/2011	60	96.67	61.75	38.25	0.00	0.00	1
23	AR	AR	27.2	535	21/06/2011	154	91.56	1.09	98.91	0.00	0.00	2
24	NR	NR	27.8	870	17/06/2011	201	88.06	99.93	0.07	0.00	0.00	2
25	AR	NR	23.4	410	17/06/2011	22	80.00	100.00	0.00	0.00	0.00	0
26	AR	NPH	27.9	740	19/06/2011	134	95.52	63.45	36.50	0.00	0.05	11
27	AR	AR	26.6	655	16/06/2011	252	75.16	24.11	75.84	0.04	0.00	2
28	AR	AR	24.5	475	16/06/2011	158	96.20	12.42	87.47	0.08	0.03	2
29	AR	AR	23.7	405	16/06/2011	260	71.92	0.00	99.66	0.00	0.34	1
30	AR	T	31.5	995	16/06/2011	193	20.73	85.98	13.86	0.00	0.16	2
31	AR	AR	24.7	525	16/06/2011	146	98.63	18.02	81.91	0.07	0.00	3
32	NR	NR	26.0	550	30/05/2011	219	94.06	100.00	0.00	0.00	0.00	0
33	AR	AR	25.3	485	30/05/2011	164	99.39	0.16	99.84	0.00	0.00	1
34	AR	NPH	18.3	205	30/05/2011	269	73.98	47.84	52.16	0.00	0.00	7
35	NR	NR	30.0	785	30/05/2011	220	95.91	99.98	0.02	0.00	0.00	1
36	NR	NR	32.4	955	30/05/2011	186	75.27	99.82	0.18	0.00	0.00	3
37	AR	T	18.5	200	28/07/2011	83	9.64	56.00	40.00	2.00	2.00	2
38	AR	AR	18.2	190	28/07/2011	102	95.10	0.00	100.00	0.00	0.00	0
39	NR	AR	32.6	1140	29/07/2011	111	93.69	2.86	97.09	0.00	0.05	1
40	NR	NR	31.4	935	29/07/2011	161	95.03	99.79	0.21	0.00	0.00	3
41	NR	NR	26.6	545	29/07/2011	117	95.73	99.11	0.89	0.00	0.00	4
42	NR	NR	20.5	335	29/07/2011	79	100.00	100.00	0.00	0.00	0.00	0
43	NR	NR	25.6	505	29/07/2011	159	73.58	99.71	0.29	0.00	0.00	1
44	NR	NR	32.2	970	29/07/2011	160	93.75	99.88	0.12	0.00	0.00	2
45	AR	AR	21.4	315	29/07/2011	35	80.00	0.00	100.00	0.00	0.00	0
46	NR	NR	35.0	1215	16/09/2011	125	76.80	98.19	1.81	0.00	0.00	9
47	NR	T	27.4	690	16/09/2011	166	28.92	99.73	0.00	0.00	0.27	1
48	NR	NR	20.0	225	16/09/2011	110	72.73	99.91	0.09	0.00	0.00	1
49	NR	NR	25.9	580	16/09/2011	109	71.81	99.94	0.06	0.00	0.00	1
50	NR	T	21.5	335	16/09/2011	77	45.45	99.64	0.00	0.00	0.36	1
51	AR	AR	25.0	525	24/10/2011	63	88.89	0.00	100.00	0.00	0.00	0
52	NR	NR	22.2	340	16/09/2011	32	93.75	99.76	0.00	0.00	0.24	1
53	NR	NR	19.6	220	16/09/2011	110	70.27	100.00	0.00	0.00	0.00	0
54	NR	NR	19.4	245	16/09/2011	54	75.56	98.48	0.38	0.00	1.14	2
55	NR	T	22.9	375	16/09/2011	26	34.62	100.00	0.00	0.00	0.00	0
56	NR	T	24.8	480	16/09/2011	110	38.18	100.00	0.00	0.00	0.00	0
57	NR	NR	23.6	420	16/09/2011	109	74.31	100.00	0.00	0.00	0.00	0
58	NR	T	24.9	290	16/09/2011	106	25.47	96.35	0.66	0.00	2.99	2
59	NR	T	17.0	155	16/09/2011	110	23.64	100.00	0.00	0.00	0.00	0
60	NR	T	22.9	395	17/09/2011	30	16.67	100.00	0.00	0.00	0.00	0
61	NR	NR	18.3	210	17/09/2011	110	95.45	99.95	0.05	0.00	0.00	1
62	AR	AR	18.2	230	31/10/2011	79	78.48	0.00	100.00	0.00	0.00	0
63	SW	T	30.3	945	20/10/2011	43	37.21	98.70	0.00	0.00	1.30	1
64	AR	NR	26.2	605	30/09/2011	29	89.66	94.04	5.96	0.00	0.00	1
65	AR	T	19.9	310	03/11/2011	29	6.90	0.00	60.00	0.00	40.00	1
66	NR	NR	32.0	975	07/09/2011	54	87.04	100.00	0.00	0.00	0.00	0
67	SW	NR	19.7	245	09/10/2011	102	74.71	99.64	0.36	0.00	0.00	1
68	NR	NR	27.9	750	07/09/2011	60	85.00	99.76	0.24	0.00	0.00	1
69	AR	AR	31.6	1010	11/09/2011	59	88.14	0.00	100.00	0.00	0.00	0
70	AR	AR	21.6	285	11/09/2011	57	94.74	0.00	100.00	0.00	0.00	0
71	AR	AR	20.1	270	11/09/2011	30	96.67	0.00	100.00	0.00	0.00	0
72	AR	T	24.0	415	11/09/2011	57	40.35	95.61	4.39	0.00	0.00	1
73	NR	NR	25.5	550	07/09/2011	26	92.31	100.00	0.00	0.00	0.00	0
74	AR	AR	31.6	885	30/07/2011	161	95.65	17.72	82.24	0.03	0.00	2

(AR: Artificial Reef, NR: Natural Reef, T: Transient fish, NPH: No-Preferred Habitat fish, DP: Detection Period, DR: Detection Rate, RI: Residency Index, SW: SeaWalls, CV: Côte Vermeille, EOPH: Excursions Outside Preferred Habitat for resident fish and outside capture location for the other fish).

### 3. Seascape connectivity between artificial and natural habitats

Out of the 74 monitored fish, 61 fish (82%) were detected at least once on the NR of Cape Leucate, 55 fish (74%) on the ARs, 7 fish (10%) on the breakwaters and 17 fish (23%) on the Côte Vermeille which is located 35 km from the ARs and the NR. Among the 53 resident fish of the NR and the ARs, 40% (n = 21) were never detected outside their preferred habitat, 26% (n = 14) were detected once, 21% (n = 11) were detected twice and only 13% (n = 7) were detected more than twice outside their habitat. The maximum number of excursions outside their preferred habitat for resident fish was nine times (Table 2).

**Figure 4:**
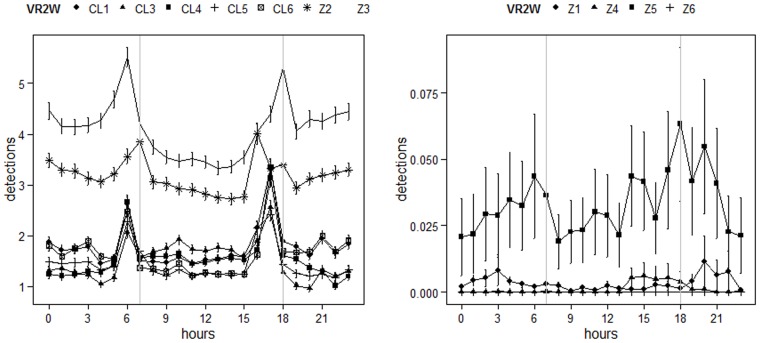
Mean detections and standard errors per hourly bin by receivers of the artificial reef (Z1–Z6) and of the natural reef (CL1-CL6). On the left side are receivers with the most detections which were visited on a daily basis by white seabreams. On the right side are the receivers which were visited episodically. Vertical grey lines symbolize mean sunset and sunrise over the entire study period, to get an approximate delimitation of day- and night-time.

Fish detected on the seawalls were either AR-residents (n = 4) or transient fish (n = 2) and one was an NPH fish; none was an NR-resident. For all fish except for one transient (fish #16), excursions to the seawalls occurred only once and never exceeded a few hours. All detections on seawalls occurred in summer (June-July). No diel pattern was visible (Fig. 4 b) for detections of receivers on the seawalls, but detections were very low and episodic. Fish detected on the CV were from all behavioral groups (AR, NR, NPH, T) and were detected in this habitat during the cold season between October and March, which was the end of the acoustic survey. Only the transient fish #16 was detected for a twelve-day period in July on the CV. None of the fish detected on the CV were subsequently detected again, with the exception of the transient fish #58, which was detected three different times by the CV receivers, in October, November and December. It was also the only fish to return to the Cape Leucate NR after being detected on the CV in the south. Receivers from the NR were all equivalently visited by tagged white seabreams, whereas strong differences were visible in detection numbers of the AR receivers (Fig. 4a, 4b). The highest detection rates were seen on the AR subsets Z2 and Z3.

### 4. Temporal movement pattern

**Figure 5:**
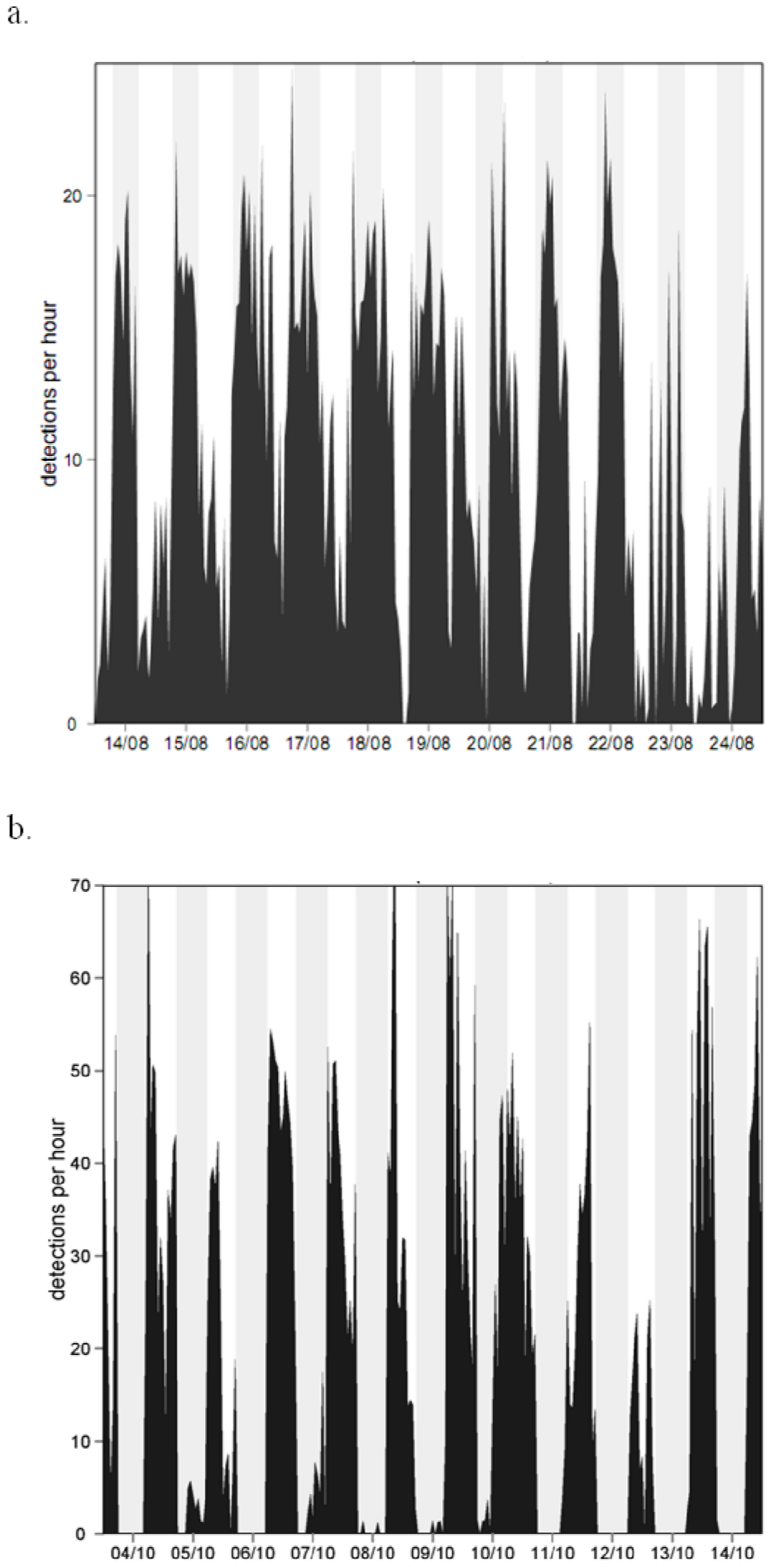
Chronogram plots of fish #39 an AR-resident (a) and fish #61 a NR-resident (b) over a ten-day period. Grey areas represent night-time.

**Figure 6:**
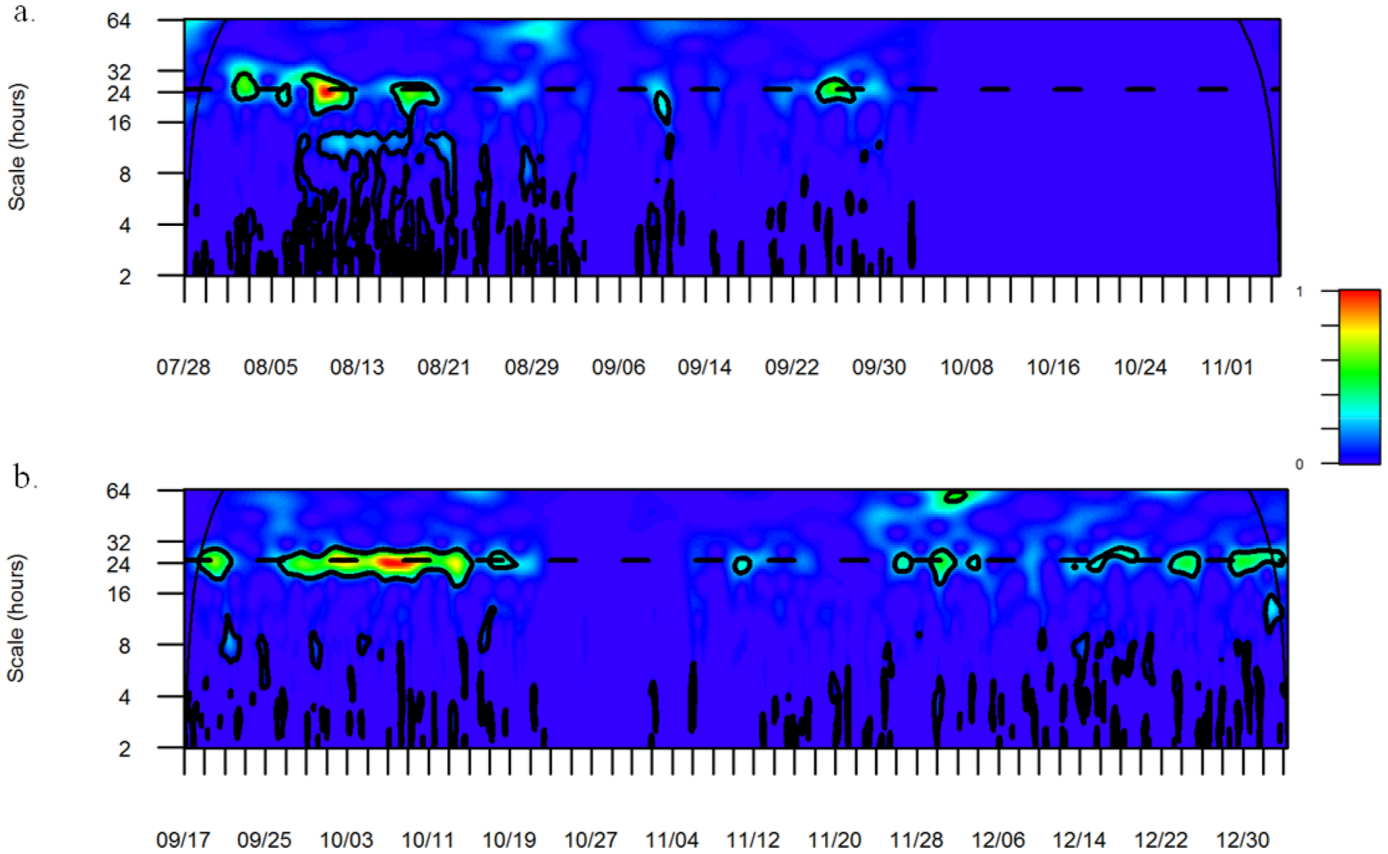
Wavelet sample spectrums of AR-resident fish #39 (a) and NR-resident fish #61 (b) over the entire detection period. Continuous lines represent the cone of influence (COI) above which data should not be interpreted. The thick contours represent the 95% confidence level and significant periodicities. Dashed line represents the 24 h periodicity threshold.

Individual chronogram plots of all tagged fish highlighted two different inverse patterns (examples of two fish over a ten-day period in Fig. 5a, 5b). The AR-resident fish (#39) showed higher detection rates at night than during the daytime, whereas the NR-resident fish (#61) showed higher detection rates during the daytime and lower during the night. For some fish, no clear temporal pattern was visible on the chronogram plots. The CWT analysis for each individual fish reported no significant temporal periodicity for fish tags over the entire study period, and only a few 24 h periodic patterns were visible over shorter periods of about a few days (examples of two fish, #39 and #61, in Fig. 6).

**Figure 7:**
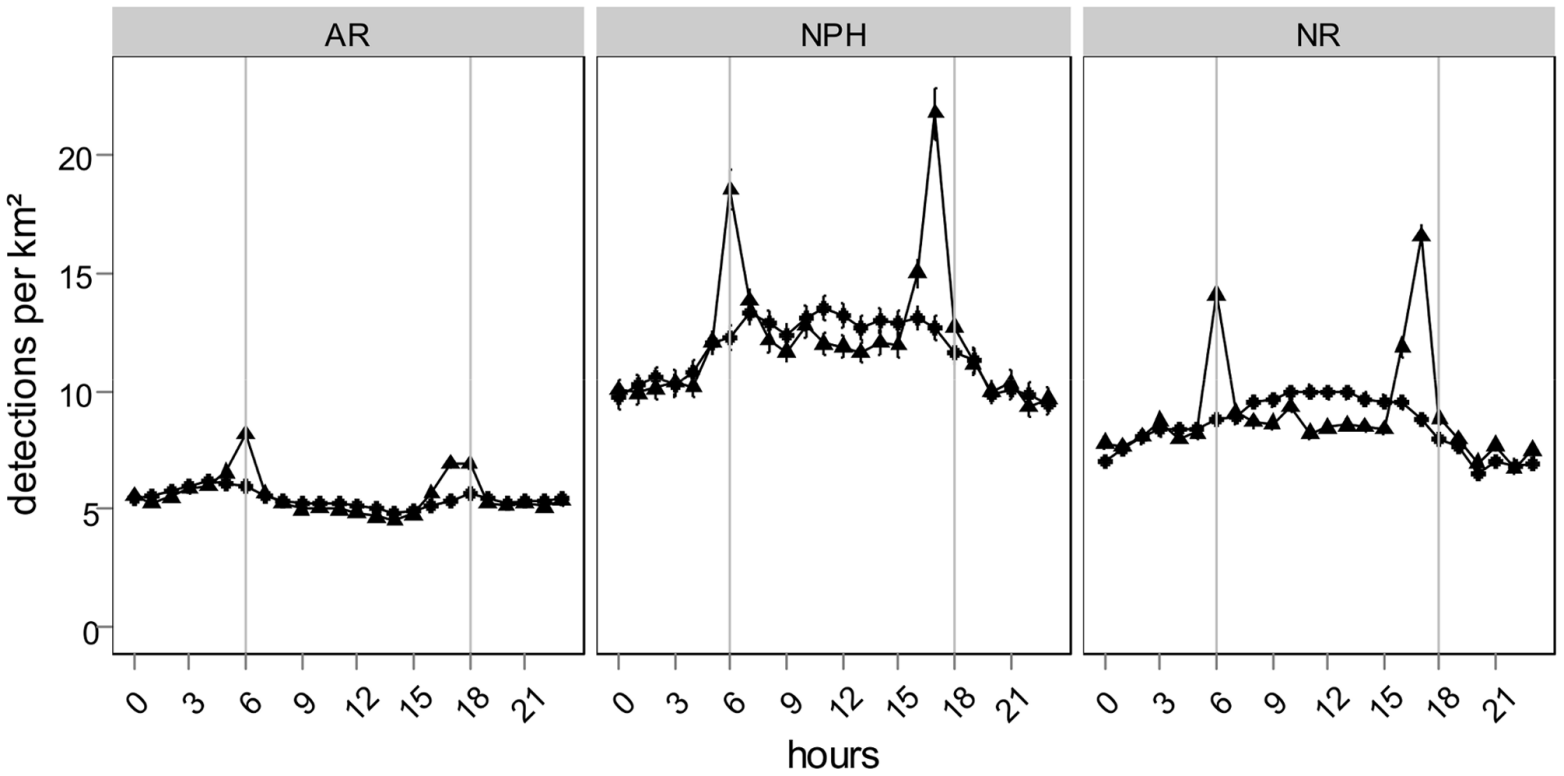
Mean detections and standard errors per hourly bin of AR-residents and NR-residents of preferred habitat and NPH fish (No-Preferred Habitat) on AR and NR pooled together. Dots represent raw detections and triangles detections corrected by control-tags. Detections are standardized by sampling surface and thus expressed per km^2^. Vertical grey lines symbolize mean sunset and sunrise over the entire study period, to get an approximate delimitation of day- and night-time.

These two inverse diel patterns were also visible when pooling all fish by behavioral group and preferred habitat (Fig. 7). Mean detections per hourly bin of the AR-residents (only the detections on the ARs were kept) seemed to be lower during the day than the night, whereas for the NR-residents (only the detections on the NR were kept) and the NPH fish they seemed to be lower by night. We noticed the difference of temporal diel patterns before and after the standardization by control tags. After the correction, differences in detections between day and night were reduced for the NPH fish and NR-residents. For all three behavioral groups, mean detections sharply increased around the dusk and dawn periods. The formal testing of detections between daytime and size of fish using a GLMM analysis showed significantly higher detections during the night on the AR and during the daylight hours on the NR (Table 3). No differences in detections were observed for size of fish, only the interaction factor of size and daytime was significant for both habitats. When comparing between detections for behavioral groups, GLMM analysis showed significant differences in detections between day and night, with higher detections during the night for the AR-resident fish and higher during the daylight hours for the NPH fish in both habitats and for the NR-resident fish (Table 4). Detections of all behavioral groups were independent of fish size, with however significant differences for the interaction terms between diel-phase and size. Only for NPH fish on NR, detections were significantly higher for smaller individuals than larger ones, but with no differences for the interaction term of diel phase and size. Comparison of fish size distribution between the different behavioral groups showed no differences (F = 0.693; p-value = 0.504).

**Table 3:** Results of generalized linear mixed models testing the effect of diel phase (day vs. night) and fish size on mean white seabream detections pooled by reef type: NR (natural reef) and AR (artificial reef).

	estimate	SE	z-value	Pr(>|t|)	
***a. NR***					
intercept	2.7060	0.3719	7.27	3.44e-13	***
diel phase (day)	0.2244	0.0118	19.01	<2e-16	***
size	0.00002	0.0013	0.02	0.984	ns
diel phase (day)* size	−0.00049	0.00004	−11.81	<2e-16	***
***b. AR***					
intercept	2.1620	0.0582	37.14	<2e-16	***
diel phase (day)	0.2687	0.0186	14.39	<2e-16	***
size	0.00001	0.0002	0.07	0.945	ns
diel phase (day)* size	−0.0012	0.00006	−17.59	<2e-16	***

‘ns’ p>0.05, ‘*’ p<0.5, ‘**’ p<0.01, ‘***’ p<0.001.

**Table 4:** Results of generalized linear mixed models testing the effect of diel phase (day vs. night) and fish size on mean detections per fish movement group and preferred habitat, i.e. detections on NR for NR-residents, on AR for AR-residents and on NR and AR separately for NPH fish.

	estimate	SE	z-value	Pr(>|t|)	
***a. NR-residents***					
intercept	2.7496	0.080	34.15	<2e-16	***
diel phase (day)	0.0965	0.002	36.98	<2e-16	***
size	−0.0179	0.076	−0.24	0.814	ns
diel phase (day)* size	−0.0306	0.002	−12.78	<2e-16	***
***b. AR-residents***					
intercept	1.9150	0.122	15.67	<2e-16	***
diel phase (day)	−0.1252	0.003	−33.28	<2e-16	***
size	−0.0024	0.119	−0.02	0.984	ns
diel phase (day)* size	−0.0856	0.004	−17.54	<2e-16	***
***c. NPH on NR***					
intercept	2.9372	0.095	30.68	<2e-16	***
diel phase (day)	0.0759	0.005	13.04	<2e-16	***
size	0.3262	0.073	4.45	8.48e-06	***
diel phase (day)* size	−0.0052	0.004	−1.17	0.240	ns
***d. NR on AR***					
intercept	2.1234	0.138	15.38	<2e-16	***
diel phase (day)	0.1381	0.007	17.89	<2e-16	***
size	0.0862	0.120	0.71	0.475	ns
diel phase (day)* size	−0.0195	0.005	−3.84	0.0001	***

‘ns’ p>0.05, ‘*’ p<0.5, ‘**’ p<0.01, ‘***’ p<0.001.

## Discussion

### 1. Need for control tags to account for environmental variability

Even if acoustic tagging has been in use for decades for the monitoring of marine animal movements [52], the deployment of control tags during the whole duration of the experiment has only recently been advocated [54]. Payne *et al.* [54] showed that the observed diel detection pattern of cuttlefish (*Sepia apama*) was solely due to fluctuating diel detection probabilities, which decreased at night due to environmental noises. Since that work, some studies have integrated control tags into their study design to validate their detection patterns [27], [64]. Diel variations of detection probabilities were notably noticed in the study of Bryars *et al.* [64], carried out on a heterogeneous coastal reef with sandy bottoms and seaweed along Kangaroo Island in South Australia.

In our study, results from the control tags showed clear environmental variation during the sunrise and sunset and slightly diel variations in detection probabilities and with different magnitudes depending on habitat type. The sharp decrease in detection rate noticed during dawn and dusk could be explained by increased biological activity. Radford *et al.* [65] showed that along a shallow rocky reef of New Zealand, the main source of noise was the biotic activity of the feeding behavior of sea-urchins and the snapping of shrimps during the night. These noises could represent up to 20 dB and were particularly high during the dusk and the dawn periods, and are known under the term “evening choruses”.

On the AR system of Leucate – Le Barcarès, the propagation of acoustic waves seems to vary less than on the Cape Leucate NR, where detection rates were significantly higher during daytime than by night. Differences in detection range and rates between the two locations may be due to habitat configuration, as the ARs are located along a sandy coast in contrast with the NR, which is a very uneven rocky habitat. Higher detection rates during daytime than night were also noticed by How and de Lestang [53] who tested several factors affecting the detection probabilities of acoustic tags and emphasized that detection rates are highly influenced by diel phase. As the factors affecting acoustic wave propagation can be numerous and difficult to monitor, the use of control tags as suggested by Payne *et al.* [54] seems to be a simple option to clear the acoustic signal from “environmental noise”. Nevertheless, it is necessary to have a good knowledge of the study area (substrate type, hydrological conditions and environmental forcing) to identify the different areas where control tags should be deployed. If this knowledge is lacking, it could be meaningful to deploy control tags spread over the acoustic receiver array. The use of control tags and the standardization of observed detections by control tag pattern would thus avoid misleading ecological conclusions concerning the movement patterns of animals.

### 2. Seascape connectivity between artificial and natural habitats

In many management and conservation strategies, species and populations are considered as homogeneous entities with stationary movement patterns, assuming that individual variability in behavior is negligible and that the landscape in which they move is homogeneous. However, recent studies have brought to light how the consideration of landscape ecology could contribute to conservation [66]. Considering seascape ecology and the behavioral diversity pattern of animals is particularly important when planning an AR project, given the physical alteration of seascape connectivity which can be expected to alter the habitat use of individuals and population dynamics by facilitating or restricting movements between habitat patches [14]. In our study we showed how ARs act on seascape connectivity and the temporal diel behavior of the white seabream, which is a widely distributed temperate-reef fish targeted by professional and recreational fisheries [67], [68], [69].

Most of the tagged white seabream (73%) showed strong site fidelity either to the ARs of Leucate –Le Barcarès or the NR of Cape Leucate. Like natural rocky reefs, these ARs seem thus to be an essential habitat for white seabream, at least where refuge and feeding are concerned. To obtain information about the habitat use of white seabream during the reproductive period, it would be necessary to monitor fish movements during the reproductive period in spring [39]. Other acoustic tagging experiments have shown the potential of ARs as suitable habitats for reef fishes in temperate [45], tropical [70] and also sub-arctic waters [20]. The number of excursions outside the preferred habitat for resident fish was very low, showing that exchanges between hard-substratum habitats of the study area exist but that the degree of connectivity is limited due to the high residency of most individuals. A potential explanation of this low seascape connectivity between habitat patches could be the high inter-patch distances compared to the daily movement ranges of white seabream. Travel distance between habitat patches has been shown to influence functional connectivity in terrestrial ecosystems [71]. In Lino *et al.* [72] tagged white seabreams moved from ARs to NRs on a daily basis but inter-habitat distances were about 500 m. This is much lower than in our area, where the minimal distance between ARs and the NR was 2 km. Another explanation could be that sandy areas were considered as non-favorable habitats where fish are exposed to higher mortality risks and act as semi-permeable barriers to their movement capacities. A similar conclusion was drawn by Turgeon *et al.* [73] for damselfish in a coral reef system using a small-scale gap-crossing experiment. In our case we noticed that the newly added habitats were nevertheless quickly colonized by white seabreams as shown in an underwater visual survey of the studied ARs [43] and that fish visibly remain on these ARs. Given our results, the Leucate – Le Barcarès ARs do not expand, but multiply the potential habitats available to white seabream, probably due to their patchy configuration and the range of mobility and perception of fish [74]. Moreover, in the debate over the “concentration vs. production” action of ARs, our results are in favor of the real production effect of ARs due to their capacity to provide food for fish populations.

Excursions to seawalls occurred interestingly only in summer whereas excursions to the CV, which is a more extended rocky coast in the south, occurred during the cold season. According to Hussein *et al.* [75], about 20% of the white seabream subpopulation from the sandy coast, including our study location, move down to the CV when the water gets colder in autumn, which is a very close estimation to our result of 23%. The absence of detections on seawalls during the winter season could be due to a general decrease in activity and excursions outside the preferred habitat when water temperatures decrease. Nevertheless, an acoustic monitoring experiment of white seabreams over an entire year would be necessary to ensure that this southward movement does not convey a winter migration pattern, with fish coming back in spring. At a smaller spatial scale, differences in habitat utilization were also seen between the different artificial subsets. These differences can be partially explained by the uneven number of fish caught and released on the different AR subsets, which was the highest on the northern AR groups Z1 and Z2 and the central reefs Z3, but not only as the fishing effort was evenly distributed between these AR groups. In fact, most visited receivers on the ARs were located at the shallowest AR subsets (Z2, Z3 and Z5) ranging between 15 and 19 m depth, with particularly high detection rates at those closest to the NR Cape Leucate (Z2 and Z3). This result highlights the bathymetric preferendum of white seabream for shallow waters above 20 m depth, but probably also a kind of connectivity to the closest NR at Cape Leucate.

### 3. Behavioral diversity in habitat utilization

The movement and habitat use patterns of reef fishes can be very complex, sometimes with high individual variability. Some fish species show clear resident movement patterns [64], [76], [77] while others have different individual movement dynamics [28], [78], [79]. In our study, we observed that the behavioral patterns of white seabream differed not only between resident and transient fish, but also in the diel habitat use of resident individuals. The temporal analysis of white seabream detections reveals contrasting diel movement patterns between habitat types (AR and NR) but also between behavioral fish groups (AR-resident, NR-resident, NPH-resident). The higher detection rates on the NR of NR-resident and NPH-fish during daytime can be linked to a diurnal pattern with increased foraging activity during the day and resting periods during the night. This inference is supported by the fact that white seabream are known to shelter in crevices during the night [80], which would have reduced the acoustic signal transmission and thus the detection rates of receivers. On the contrary, the higher detection rates on the AR for AR-resident fish can be linked to a nocturnal pattern with higher activity by night and resting time during the day. Such a pattern has been recently reported by D'Anna *et al.* [45], where the authors carried out a fine-scale receiver array which permitted them to highlight that fish were hiding inside the AR set during the day and searching for food by night around the artificial structures on sandy- and seaweed-covered bottoms. However, fish that switched between the ARs and NR during the study period (NPH behavioral pattern) showed a mainly diurnal pattern, like the NR-resident fish, regardless of the habitat type. Despite the control tag corrections of detection patterns across habitats, the analysis of acoustic detection frequencies remains an indirect method of observing diel movement patterns. The fact that the NPH fish remained diurnal across habitats confirms thus the presence of opposite diel movement patterns within the resident white seabreams and asserts that these findings are not consistent with environmental detection artifacts. Furthermore, no differences in size distribution between behavioural groups could be observed, confirming that the presence of two opposite diel movement patterns is not due to a simple sampling bias (e.g. hypothetically, if more small individuals were sampled on ARs than on NR).

While diel movement patterns exhibited by white seabreams were independent of size, the interaction between diel phase and size was significant for nearly all fish groups (NR- and AR-resident, NPH fish on NR), with larger fish being less detected the day. A possible explanation could be that larger fish were either better hidden under rocks or in crevices during resting periods (in case of a nocturnal pattern) or less active or active over shorter periods than smaller white seabreams during foraging periods (in case of a diurnal pattern). The only exception was the detection pattern of NPH fish on NR, which were related to size, with small individuals displaying more detections than larger fish. Due to the fact that the NR Cape Leucate is the only natural rocky reef in the area and is of small extend, competition pressure could be very important on this location. Smaller individuals, which are not “full-time resident” on the NR, could be outcompete by larger individuals or NR-residents and have thus to travel across larger foraging areas to cover their energetic budget. The smaller NPH fish could also correspond to previous NR-residents, which at adult size were outcompete by other larger individuals on the NR due to high intra-specific competition, and were driven to explore other habitats.

In their review, Bolnick *et al.* [81] highlighted that significant inter-individual variation in niche use can occur even within sex or age. Moreover, a majority of the reported cases of individual specialization concerned fish species [82]. Even if these two diel behavioral patterns have already been reported, our study shows that diurnal and nocturnal behaviors co-occur. The expression of one or the other habitat use pattern varies depending on habitat complexity and configuration (i.e. between AR and NR), and behavioral fish group, arguing for individual variation in habitat use. A possible explanation for these two opposite behaviors could be that fish are able to adapt their behavior to their habitat according to the optimal foraging [83] and niche variation theory [84]. The resource choice of individuals is expected to be directed by the optimization of their cost/benefit ratio in order to maximize net energy income or reproductive success, which depends on resource availability and quality, but also on risks like competition and predation pressure.

Even if ecological interactions like competition and predation have not been directly measured on the two studied habitats, some plausible assumptions can be made. Previous underwater visual counts have displayed much higher fish densities on the ARs than on the NR [43], with notably particularly high concentrations of the European conger (*Conger conger*), a nocturnal predator of white seabreams. The higher tridimensional structure of ARs compared to the NR, would support a higher amount of refuges than on the NR, explaining the observed differences in fish densities and community structure. If the ARs are good refuges for the white seabreams, the high nocturnal predation pressure exerted by the congers could explain the shift of AR-resident white seabreams from a diurnal to a nocturnal behavioral pattern. Given the patchy configuration and small size of the studied ARs, this behavioral shift could also be due to resource competition, leading AR-residents to change their foraging strategy and preys to nocturnal sand-dwelling invertebrates. Such process has been described in other fish species like the European perch (*Perca fluviatilis*), whose largest young-of-the-year undergo a diet shift from zooplankton to macroinvertebrates under strong intra-specific competition [85]. Hammerschlag-Peyer and Layman [79] showed that movement metrics identified by acoustic monitoring can be significantly related to the resource use of coastal fish, emphasizing individual specialization. White seabream presented strong site fidelity to artificial and natural reefs, demonstrating that these two habitats provide a suitable habitat, even if they differ in structure and configuration and probably thus also in resources, resource availability, and in competition and predation risks. The diurnal and nocturnal behavioral patterns could be the expression of individual specialization resulting from habitat differences between artificial and natural reefs. Further research is necessary to confirm this hypothesis of intra-population niche variation, notably to see if AR-resident and NR-resident fish do indeed have different resource use. If this hypothesis is proved to be valid, it would have important implications for ecological and conservation ecology [81], [86], highlighting the necessity of incorporating intra-population niche variation into future management plans. For example, the intra-population niche variation could result in differences in fitness [87] or vulnerability to fishing pressure. Along our study location, Lloret and Planes [88] have in fact shown a lower condition for white seabreams captured along the sandy French Catalan coast than for white seabreams captured along the southern rocky coast (Côte Vermeille). In conclusion, as artificial habitats (reefs and seawalls) have the potential to alter seascape connectivity but also individual behavioral patterns of fishes, their incorporation into future management plans of coastal areas and fisheries resources seems essential.
